# Daily Weather Conditions and Anticipated Death from Cancer

**Published:** 2018-04

**Authors:** Young Saing KIM, Dong Kyun PARK, In Cheol HWANG, Hong Yup AHN

**Affiliations:** 1. Dept. of Internal Medicine, Gil Medical Center, Gachon University, Incheon, South Korea; 2. Dept. of Family Medicine, Gil Medical Center, Gachon University, Incheon, South Korea; 3. Dept. of Statistics, Dongguk University, Seoul, South Korea

**Keywords:** Anticipated death, Cancer, Palliative care, Weather

## Abstract

**Background:**

The effect of weather conditions on human health has been documented. However, the role of daily weather on anticipated death remains unclear. This study aimed to evaluate the relationship between daily weather conditions and death in terminally ill cancer patients.

**Methods:**

We retrospectively searched a medical database of 935 consecutive terminally ill cancer patients who passed away in the palliative care unit from Oct 2009 to Sep 2013. We used Poisson regression to assess the relative risk (RR) of anticipated death based on various weather variables, using mean values calculated from the 10 d around the time of death.

**Results:**

The final study population consisted of 745 patients with a mean age of 65.9 ± 12.2 yr. The mean palliative prognostic index (PPI) score in the last week of life was 8.0 ± 3.8. After adjustment for age, sex, cancer type, and PPI score, RRs were 1.69 (95% CI, 1.17–2.46) for low temperature, 2.57 (1.77–3.77) for high diurnal temperature variation, 2.35 (1.61–3.36) for low humidity, and 1.75 (1.22–2.53) for high levels of sunlight.

**Conclusion:**

Weather conditions may be a predictor of death in terminally ill cancer patients.

## Introduction

A large body of literature is devoted to the impact of climate on the human health, as specific climate conditions can increase mortality (1, 2). For example, heat waves and cold exposure are associated with an increased number of deaths in the general population (3, 4). The elderly and patients with underlying disease are considered to be at higher risk for climate-related mortality (2). Unlike in climatology research, outcome measures in studies of weather-related mortality have been limited to sudden deaths, including those caused by cardiovascular disease and respiratory disease (5, 6). Cancer is undoubtedly the leading cause of death worldwide, of which the prognostication is a relatively lesser challenge (7). Regrettably, there have been no studies that examine the effect of weather on this anticipated death. Terminally ill patients are more susceptible to weather conditions since their physiologic capability to adequately respond to them is compromised (2, 8).

Predicting impending death in terminally ill patients is important for patients and their families since it helps them prepare deaths, facilitates the ability to carry out a patient’ desires and allows the patient to say goodbye to loved ones. Predicting a patient’s death is also crucial for medical teams in a hospital setting, allowing them to provide better service and plan bed availability. Although a number of studies on survival prediction in terminally ill patients have been performed, it remains difficult to achieve accurate estimates (9). If there is any association between weather conditions and anticipated death, it might be helpful for palliative care providers to prognosticate because the weather can generally be accurately forecast in advance.

The purpose of this retrospective study was to evaluate the relationship between daily weather conditions and death in terminally ill cancer patients.

## Materials and Methods

### Study population

We retrospectively analyzed the medical records of 935 consecutive terminally ill cancer patients who passed away in the palliative care unit of Gachon University Gil Medical Center (Incheon, South Korea) between Oct 2009 and Sep 2013. A terminally ill cancer patient was defined as someone with progressive, advanced cancer for whom conventional anticancer therapy was no longer indicated. Demographic and clinical characteristics of patients were collected by an experienced palliative care team of physicians and registered nurses. Information gathered included age, sex, primary cancer site, performance status (PS), date of admission to the palliative care unit, date of death, and palliative prognostic index (PPI) score. The PPI is a validated scoring system for survival prediction in hospice patients with advanced malignant disease that relies on patient characteristics such as PS, oral intake, the presence of edema, dyspnea at rest, and delirium (10, 11). A Korean version of the PPI was assessed weekly in our palliative care unit, and we used the last PPI scores in our analyses.

### Weather information

Our institution is located in Incheon Metropolitan City, which occupies a wide area of South Korea’s northwestern coast and many nearby islands. Korea is geographically situated in a temperate climate zone at medium latitude and has four distinct seasons. In general, spring is from Mar to May, summer from Jun to Aug, autumn from Sep to Nov, and winter from Dec to Feb. The weather in spring and autumn is clear and dry due to the influence of anticyclones; summer is hot and humid due to Korea’s location on the North Pacific Edge; and in winter, continental high pressure brings cold and dry weather. Weather data throughout the study period were measured at Incheon International Airport (latitude: 37°28′N, Longitude: 126°37′E, elevation: 69 m) and provided by the Korea Aviation Meteorological Agency. They included daily measurements of temperature (°C), temperature range (°C), relative humidity (%), and amounts of sunshine (MJ/m^2^) as well as 10-day averages. We categorized weather conditions at the day of death as “above the mean” or “below the mean” using 10-d lag (lag 0 to lag 9) average values as a reference: low *vs.* high for temperature and humidity; small *vs.* large for diurnal temperature range; little *vs.* much for sunshine amount.

### Statistical Analysis

To assess the association between weather conditions and death, Poisson regression models were employed to estimate relative risk (RR). Statistical significance was accepted for *P*-values <0.05. All analyses were performed using SAS ver. 9.2 (SAS Institute Inc., Cary, NC, USA).

### Ethical approval

The relevant Institutional Review Board approved this retrospective study. Requirement for informed patient consent was waived since this study involved only chart review, no identifying patient information was collected, and data derived from routine evaluation practices. Other ethical issues, including plagiarism, misconduct, data fabrication and/or falsification, double publication and/or submission, redundancy, etc. have been completely observed by the authors.

## Results

After excluding 190 patients lacking data on PPI (n = 188) or climatic (n = 2), the final study population consisted of 745 patients. Patient’ demographic and clinical characteristics are summarized in Table 1.

**Table 1:** Characteristics of participants

***Variable***		***No. (%)***
Number		745
Age (yr), mean±SD		65.9 ± 12.2
Female		308 (41.3)
Cancer site		
	Gastrointestinal	175 (23.5)
	Hepatobiliary	199 (26.7)
	Lung	189 (25.4)
	Genitourinary	85 (11.4)
	Other^a^	97 (13.0)
PPI score^b^, mean±SD		8.0 ± 3.8
Seasonal deaths		
	Spring	246 (33.0)
	Summer	82 (11.0)
	Fall	189 (25.4)
	Winter	228 (30.6)
*Weather conditions at the day of death*		
Temperature		
	< average_10_	373 (50.1)
	≥ average_10_	372 (49.9)
Diurnal temperature range		
	< average_10_	370 (49.7)
	≥ average_10_	375 (50.3)
Humidity		
	< average_10_	345 (46.3)
	≥ average_10_	400 (53.7)
Sunshine		
	< average_10_	312 (41.9)
	≥ average_10_	433 (58.1)

SD = standard deviation; PPI = palliative prognostic index. //Average_10_ refers to a mean value of 10 d near death.

a Includes breast cancer, hematologic malignancies, *etc*. //

b From last week of life.

Mean age at death was 65.9 ± 12.2 yr, and 58.7% of patients were male. The most common primary cancer sites were hepatobiliary system (26.7%), lungs (25.4%), and gastrointestinal tract (23.5%). The lowest proportion of deaths was observed in summer, with 33.0%, 11.0%, 25.4%, and 30.6% of deaths occurring in spring, summer, autumn, and winter, respectively.

In light of the potential cumulative and delayed influence of weather on the human body, we evaluated average values of weather variables during 10 d near the time of deaths. The average values of weather conditions were as follows; 11.8±10.1 °C for temperature, 6.9±1.3 °C for diurnal temperature range, 69.9±11.7% for humidity, 12.8±4.4MJ/m^2^ for amounts of sunshine. After dichotomizing mean 10-d values near death, we found that low temperature, high diurnal temperature change, low humidity, and high sunlight levels were significantly associated with death in terminally ill cancer patients (Table 2). After adjustment for age, sex, cancer type, and PPI score, the RRs were 2.17 (95% CI, 1.48 to 3.18) for low temperature, 2.53 (95% CI, 1.74 to 3.70) for large diurnal temperature variation, 2.20 (95% CI, 1.52 to 3.20) for low humidity, and 1.83 (95% CI, 1.27 to 2.64) for much sunshine.

**Table 2:** Relative risk^a^ of death associated with various weather characteristics

		***Unadjusted***		***Adjusted***	
Weather condition^b^	No.	RR (95% CI)	*P*	RR (95% CI)	*P*
Low temperature	373	1.97 (1.65 to 2.36)	<0.0001	2.17 (1.48 to 3.18)	0.0001
Large diurnal temperature	375	2.05 (1.71 to 2.45)	<0.0001	2.53 (1.74 to 3.70)	<0.0001
Low humidity	345	1.89 (1.58 to 2.28)	<0.0001	2.20 (1.52 to 3.20)	<0.0001
Much sunshine	433	1.60 (1.32 to 1.93)	<0.0001	1.83 (1.27 to 2.64)	0.0012

RR = relative risk; CI = confidence interval.

a Derived from Poisson regression models with or without adjusting for age, sex, cancer type, and the last palliative prognostic index score. //

b Based on the mean value of 10 d near death.

## Discussion

This preliminary study investigated the impact of weather conditions on anticipated death. Previously, weather may trigger some types of deaths, providing an explanation for temporal clusters (12). In this study, weather conditions were associated anticipated death in terminally ill cancer patients.

The association between weather and anticipated death in terminally ill patients is generally overlooked and has not yet been evaluated in the literature. This is because of insufficient awareness of human biometeorology and the absence of a demonstrated causal connection (13). Determining the potential impact of weather on mortality in cancer patients is complex due to numerous confounding factors related to cancer death (14). Despite these challenges, this is the first study to report that death in critically-ill cancer patients is associated with weather conditions around the time of death; low temperature, large diurnal temperature fluctuation, low humidity, and much sunshine were statistically significant risk factors for death.

One advantage of our study is the homogeneity of the data. Our results were based on data collected from cancer patients in a single palliative unit, which may be beneficial for minimizing confounding factors, such as weather differences between microenvironments and institutional variations in care. Strength is the simplicity of our methods, which may be easily applicable to in other institution settings. To explore the association between weather variables and anticipated death, 10-d average values were analyzed. This took into account the delayed physiological response to weather conditions; in contrast to previous studies that focused on sudden weather changes on dates of health events, lag or cumulative effects were important considerations when assessing weather-related morbidity and mortality (15–17).

There are several potential explanations for the association between weather conditions and death. For example, cold weather induces vasoconstriction and increases blood pressure via stimulation of the sympathetic nervous system (18). Low temperature also promotes thrombosis by increasing plasma clotting factor concentration and blood viscosity (19). According to postmortem examinations, thrombosis and hemorrhage are among the principal causes of death in cancer patients: malignancy is itself a hypercoagulable condition and terminally ill cancer patients are usually confined to bed (14, 20, 21). Thus, low temperature could increase risk of thrombosis and hemorrhage, and hence death. In addition, terminally ill cancer patients may not have the physiologic capability to adequately respond to temperature changes, due to disseminated disease and related multi-organ dysfunction. Finally, the negative association between sunshine and cancer death could be related to the role that sunshine plays in serotonin production. Sunshine stimulates the production of serotonin by the brain (22), and serotonin levels in the brain increase significantly during the process of dying (23). To clarify the underlying mechanisms, further prospective study examining physiologic responses to various weather conditions is warranted.

Our study has several limitations. First, it was conducted on cancer patients admitted to a palliative care unit at single institution over a relatively short period. Thus, the presence of selection bias cannot be excluded, and our findings may not be generalizable to home-based hospice care. Second, our analyses were based on the outdoor weather data, though terminally ill cancer patients spend most of their time indoors. However, indoor climate conditions, including temperature and humidity, were positively associated with outdoor climate conditions (24).

## Conclusion

The weather conditions could be a predictor for death in terminally ill cancer patients. Understanding the effects of weather on death may help patients and physicians better anticipate and manage death in terminally illness. Additional comprehensive research is needed to affirm the association reported here and to investigate the biological mechanisms responsible.

## Ethical considerations

Ethical issues (Including plagiarism, informed consent, misconduct, data fabrication and/or falsification, double publication and/or submission, redundancy, etc.) have been completely observed by the authors.
